# Newcomers in a hazardous environment: a qualitative inquiry into sex worker vulnerability to HIV in Bali, Indonesia

**DOI:** 10.1186/1471-2458-14-832

**Published:** 2014-08-11

**Authors:** Pande Putu Januraga, Julie Mooney-Somers, Paul R Ward

**Affiliations:** School of Public Health, Udayana University, Gedung PSIKM FK Universitas Udayana, Jl. PB Sudirman, Denpasar, Bali 50232 Indonesia; Discipline of Public Health, Flinders University, Level 2, Health Science Building Flinders Drive, Bedford Park, Adelaide 5042 Australia; Centre for Values, Ethics and the Law in Medicine, University of Sydney, Level 1 of the Medical Foundation Building, 92-94 Parramatta Road, Camperdown, Sydney Australia

**Keywords:** Sex work, Newcomers, Social network, Social support, HIV prevention knowledge, Trust

## Abstract

**Background:**

Women new to sex work and those with a greater degree of mobility have higher risk of HIV infection. Using social capital as a theoretical framework, we argue that better understanding of the interactions of micro-level structural factors can be valuable in reshaping and restructuring health promotion programmes in Bali to be more responsive to the concerns and needs of newcomer and mobile female sex workers (FSWs).

**Methods:**

We conducted interviews with 11 newcomer FSWs (worked < six months), 9 mobile FSWs (experienced but worked at the current brothel < six months), and 14 senior FSWs (experienced and worked at current brothel > six months). The interviews explored women’s experience of sex work including how and why they came to sex work, relationships with other FSWs and their HIV prevention practices.

**Results:**

A thematic framework analysis revealed newcomer FSWs faced multiple levels of vulnerability that contributed to increased HIV risk. First, a lack of knowledge and self-efficacy about HIV prevention practices was related to their younger age and low exposure to sexual education. Second, on entering sex work, they experienced intensely competitive working environments fuelled by economic competition. This competition reduced opportunities for positive social networks and social learning about HIV prevention. Finally, the lack of social networks and social capital between FSWs undermined peer trust and solidarity, both of which are essential to promote consistent condom use. For example, newcomer FSWs did not trust that if they refused to have sex without a condom, their peers would also refuse; this increased their likelihood of accepting unprotected sex, thereby increasing HIV risk.

**Conclusions:**

Public health and social welfare interventions and programmes need to build social networks, social support and solidarity within FSW communities, and provide health education and HIV prevention resources much earlier in women’s sex work careers.

## Background

### HIV infection in Indonesia

In 2000, global communities took a historic step to declare and acknowledge the importance of an effective response to HIV-AIDS by placing it in the context of the broader development agenda of Millennium Development Goals (MDGs). One of many health targets was to achieve a 50% reduction in the sexual transmission of HIV. A recent UNAIDS report demonstrates that Indonesia is not on track to reach this target [1]. Worryingly, the report found condom use by FSWs and their clients had actually decreased from 68% in 2009 to 58% by 2012 [2].

In Asia and Africa, commercial sex work is a key driver of the HIV epidemic, making it a major focus of research and policy work [3, 4]. A national study estimated there were more than 200,000 female sex workers (FSWs) in Indonesia, with almost 15,000 of them living with HIV [5]. In 2007, the Indonesian government implemented a national HIV strategy in response to the HIV epidemic and MDG targets. In 2011, this strategy was revitalized and new programmes to build FSW empowerment were seen as an integral component of HIV prevention [6]. With this policy landscape as the backdrop, this paper explores the HIV prevention practices of FSWs who are ‘new to the business’ in order to understand the impacts of the policy and highlight areas for further action.

International reports on the Indonesian HIV epidemic usually present rates for the country as a whole. This practice masks geographical differences in HIV rates in a country with a population of almost 240 million people (4th largest in the world), over 13,000 islands and 33 provinces [4, 7]. It also hides the different contexts of FSW environments that promote or inhibit condom use and other HIV prevention practices. Uncovering how and why FSW environments in particular areas impact on HIV prevention practices is critical in order to develop and deliver appropriate and targeted interventions, but there is very little evidence on this to date.

One area with high rates of HIV infection among FSWs is Bali Province. Annual serological surveys conducted by the Bali Health Office since 2000 show high rates of HIV infection among brothel-based and other FSWs. Brothel-based FSWs have experienced the highest increase, from 0.62% in 2000 to 20.16% in 2010 [8]. This high prevalence is accompanied by a high prevalence of gonococcal and chlamydial infections, both important factors in the high risk of HIV infection among brothel-based FSWs and clients [9]. Moreover, behavioural surveys in 2009, 2010 and 2011 show that consistent condom use among brothel-based FSWs remains below 40%, much lower than the national target to achieve a minimum 60% consistent condom use among high risk groups including FSWs [6].

Despite clinical and behavioural interventions such as regular STI diagnosis and treatment, and socially-oriented programmes, such as FSW empowerment via peer education, the rate of HIV in Bali is increasing while the rate of condom use remains low. This article explores these problems, particularly in relation to newcomer FSWs. We will argue that these women face much higher levels of vulnerability because of their lack of a ‘feel for the game’ [10]. Our paper provides an understanding about how social and environmental factors interact to influence the continuation of the risky behaviour of FSWs, and we argue that such evidence is required to reshape and restructure appropriately current programmes to be more responsive to HIV prevention problems, particularly in relation to newcomer FSWs.

### The context of mobility of FSWs in Bali

Mobility of FSWs has long been known as factor contributing to the spread of HIV [11–14]. A study to understand mobility, risk patterns, and HIV/STI rates of FSWs in different entertainment venues in China found that FSWs in higher risk venues were more mobile than those from other establishments [11]. Similarly, two studies in India found that FSWs with greater mobility had unprotected sex with clients more often and for more money than FSWs with less mobility [12, 13]. Furthermore, a qualitative study among formerly trafficked women currently engaged in sex work and their service providers on the Mexico–US border found that circumstances resulting from migration and entry to sex work shaped vulnerability of women to HIV infection [14].

Meanwhile, in Bali the importance of sex workers’ mobility to the outcomes of HIV prevention programmes was recognized years ago [3, 15–17]. The majority of FSWs in Bali are not Balinese, with most having migrated from East Java, the province closest to Bali [15, 18]. An evaluation study of peer education programmes in Bali in 1998 reported that only 50% of peer educators were still working in the clusters where they were trained one month after peer education training [16]. Furthermore, the Bali AIDS Study reported that FSWs’ mean duration for working in Bali was 13 months with a median of 6 months [3], highlighting the transient and migratory nature of FSWs in Indonesia. The study found that high turnover of FSWs in locations influenced the coverage and outcomes of HIV programmes, which relied on health educations sessions and condom distribution by fieldwork staff from a local non-government organization (NGO) along with STI diagnosis and treatment at local clinics. The study found women who were relatively new to sex work (less than 6 months) had markedly higher levels of STIs and much less knowledge of HIV-AIDS and other STIs than women who had been working for longer [3]. More recent data from a national biological survey of HIV prevalence among high risk groups found the proportion of brothel-based FSWs in Bali infected in their first six months of sex work (15%) was significantly higher than the proportion infected after working for longer than six months (12%) [19].

These epidemiological studies do not provide insights into the reasons for increased HIV vulnerability among newcomers, or provide a useful direction for targeted interventions. Our article offers a detailed understanding of the complex interaction of social and environmental factors underpinning the relationship between FSW mobility and risky behaviours in newcomer FSWs in Bali.

### Theoretical framework for analysis of social factors

To analyse how FSW mobility and social factors interact to influence newcomers’ vulnerability to HIV, we used the concept of social capital. This is a relatively recent construct in public health literature, becoming more popular since critiques of epidemiology’s inability to capture social phenomenon underlying health outcomes [20]. There are a number of definitions of social capital but for those working at the community level, Bourdieu’s conceptualization is most popular [21–24]. Bourdieu [25] conceptualized social capital as “the aggregate of the actual or potential resources which are linked to possession of a durable network of more or less institutionalized relationships of mutual acquaintance and recognition” [18], that is, as resources accrued by individuals. In the context of FSWs in Bali, social capital becomes a resource in the social struggles which shape their identity, opportunities and risks [26]. In this paper, we particularly explore the structural and cognitive aspects of social capital. Structural social capital is defined as membership of and participation in FSW groups and activities, while cognitive social capital is the trust (confidence and faith) individual FSWs have in other FSWs on important issues related to their work, such as condom use and STIs. Cognitive social capital also relates to perceived feelings of social support and reciprocity, that one FSW can go to another FSW for help or support when in need, and can count on someone in times of need. By exploring issues related to psychosocial and social factors within the Bali context, we provide a unique adaptation of Bourdieu’s approach to social capital to explain the health inequalities of women new to sex work.

There have been a few efforts to strengthen social capital and examine the effects of this on vulnerability to HIV [27]. A review of literature linking social and structural determinants of HIV/AIDS found that a better understanding of the relationship between social capital and HIV risk could influence and inform prevention activities [28]. Studies analysing social capital and sexual health among groups of teenagers in Africa found psychosocial attributes such as knowledge about HIV/AIDS, perceived personal vulnerability and self-efficacy led to HIV avoidance [29]. In a given cultural, socio-economic and epidemiological context, these psychological factors are significantly and positively influenced by the presence of social capital in the community [29, 30]. The authors of a study of FSWs and men who have sex with men in India argued that the positive association between social capital (group cohesion, social support and self-efficacy) and consistent condom use supported the development of interventions to increase social capital as an HIV prevention strategy [31].

Studies on social capital and sexual health, particularly HIV prevention practices, have been dominated by quantitative approaches and so have failed to explore the underlying context, constructs and mechanisms that link social capital and health practices or outcomes. That is, they have failed to make social capital a meaningful locally-based concept [27]. Exploratory, bottom-up qualitative inquiries are needed before we can apply the concept of social capital in epidemiological and intervention-based studies [21, 32–34].

## Methods

### Research setting

Despite sex work in Bali being illegal, brothel locations or complexes (*lokasi* or *komplek*) are easily identifiable. Mainly located close to harbours or infrastructure developments where migrant workers stay and work [35], they often consist of a large gated compound with a number of huts (*Wisma*). Each *wisma* is managed by a pimp(s) and comprises a greeting area, small bar (*Warung)* selling beers and bedrooms where a number of FSWs work and/or live. We collected data in eight locations in Denpasar (capital city of Bali) and two locations in Badung (highly populated area in Bali), with each location consisting of 30–110 FSWs working in several *Wisma*. We selected these locations because they represent a high concentration of sex workers and the brothels are identifiable and accessible.

### Sample

In-depth interviews were conducted among 34 FSWs from 10 brothel complexes in two districts in Bali from January to May 2013. In each complex, the selection process considered the representation of age, working duration at that brothel, participation as a member of any FSW support group and number of FSWs in each brothel. There were three groups of respondents involved in this study. The first group consisted of 11 FSWs who were new to sex work (less than six months), referred to as ‘newcomers’ in this article. The second group comprised nine FSWs who had worked at the current brothel for less than six months but had worked at other brothels previously, referred to as ‘mobile’ FSWs. The last group was FSWs who had worked at the current brothel for more than six months, referred to as ‘senior’ FSWs. While this paper focuses on newcomers to sex work, we also draw on the interviews with mobile and senior FSWs to provide different insights that contextualize and differentiate the experiences and perspectives of newcomers. Participants’ socio-demographic data is presented in Table 1.

**Table 1:** **Summary of interview participants’ characteristics**

Characteristic	Classification	Number of respondents recruited
Age in years	<25	9
	25–39	20
	> = 40	5
Time as sex worker	Newcomer, worked less than 6 months as sex worker	11
	Experienced and worked at current brothel more than six months	14
	Experienced and moved to the current brothel less than 6 months ago	9
Education	Higher/secondary high	4
	Secondary junior	11
	Primary	17
	Didn’t graduate from primary/never went to school	2
Membership in support groups	Yes	4
	No	30
Housing	In brothel	13
	Outside brothel	21

### Data collection and analysis procedure

Recruitment of study participants was facilitated by staff of a local NGO. They identified potential participants in brothels who were personally contacted at their workplaces by the first author, a Balinese public health physician with experience of working with FSWs to deliver HIV prevention programmes. Before being interviewed, they were provided with information about the study and invited to participate. Because the in-depth questions covered issues that might be considered sensitive and private, participants were offered the opportunity to be interviewed in their own working room or in a private room at an NGO office in Denpasar. All respondents chose the latter option. Open-ended, semi-structured interviews covered topics including how and why they became FSWs, the nature and extent of their social networks with FSWs, their trust in clients, pimps and other FSWs, perceptions of reciprocity between FSWs, HIV knowledge, and perceptions of their self-efficacy. Interviews also covered more specific questions relating to sexual practices and HIV prevention practices.

All interviews were recorded using a digital audio recorder and were transcribed by the first author using the verbatim method. Transcriptions were uploaded into NVivo software, version 10, where a comprehensive process of data coding and identification of themes was undertaken. Thematic framework analysis was used in order to sift, chart and sort data in accordance with key issues and themes, using five steps: familiarization; identifying a thematic framework; indexing; charting; and mapping and interpretation [36, 37]. The purpose of the analysis was to identify the form and nature of dimensions of social capital within the sex work field of brothels in Bali (contextual analysis) and to examine how these social factors could explain the high vulnerability of the newcomers to HIV (diagnostic analysis). The presentation of the analysis includes many quotations from interviews to make the paper more descriptive of FSWs’ experiences. All data capture, transcription, coding and analysis were conducted in the Bahasa Indonesia language to retain the original cultural and social meanings and not to lose nuanced or complex meanings that may occur during translation into English. Quotations for publication purposes were translated into English by first author.

### Ethical considerations

Ethical approval was granted by the Social and Behavioural Research Ethics Committee at Flinders University in Adelaide, Australia (number 5913 SBREC) and the Institutional Review Board of *Yayasan Kerthi Praja* in Bali, Indonesia (number 040/YKP-IRB/2012). Sex work is illegal in Indonesia and asking participants to sign a consent form could expose them to prosecution. Instead, they provided verbal consent. We also provided both written and verbal assurances to FSWs about protecting their anonymity. Names used in the quotes or text in this paper are not participants’ real names. Participants received a small payment (100,000 Rupiah = $10 AUD) to compensate for their time. Furthermore, in accordance with BioMed Central editorial policies for reporting qualitative studies, we hereby stated that this article has adhered to the RATS guidelines.

## Results

Data analysis revealed three core themes associated with the HIV vulnerability of newcomers: HIV knowledge, experiences and self-efficacy; competitive working environment of brothel-based sex work; and lack of social capital in sex work communities. These three core themes create a linked and potentially cumulative network of factors influencing newcomer FSWs’ vulnerabilities to HIV.

### Lack of knowledge, experiences and self-efficacy related to HIV prevention practices

As described earlier, the majority of FSWs in Bali were migrant workers from other provinces and islands in Indonesia. While some of the women were experienced sex workers from other brothels in those areas, most FSWs originally came to Bali seeking socially acceptable work. They were aiming for success, but because of their low educational background (see Table 1) and lack of skills for well-paid work, they had to accept sex work. Two FSWs, Kanti a newcomer and Juni a senior worker, describe their goals:

- Kanti:
- A: Once I worked at a grocery, but the salary wasn’t enough. When my kid called for money and you only get seven hundred fifty (in thousand rupiah, $1 US equal to 11,000 Rupiah), how was that enough?
- Juni
- A: I would like to become as successful as my neighbour in my village. I want to have land and a good house.
- Q: Have you seen someone, a sex worker, become that successful?
- A: Don’t know, never found one yet, but I will try my best to work here. I will work hard for money.

Unfortunately, these women not only lacked the education and work skills for more socially acceptable work, they also entered sex work with little knowledge or skills about HIV prevention practices. A number of behavioural surveys in Indonesia have shown sub-optimal levels of knowledge about the protective benefits of condoms among brothel-based FSWs [7, 19, 38]. Our study indicated knowledge levels among newcomers are even worse. One senior FSW suggested this was because of the lack of experience among the predominantly young newcomer FSWs:

- Ayu:
- A: [It] depends on what kind of newcomer, the real one or the other kind of new. The real newcomers, mostly they are young and innocent, they don’t know what they are dealing with.

Among our participants, newcomers’ inadequate level of knowledge and experience of STIs and HIV prevention seemed attributable to their age and education. The majority (8/11 FSWs) were young (18–25 years), had a low level of education, and had no school or family based education on sexual and reproductive health. Our interview data suggest they entered sex work with very little, if any, knowledge of STI or HIV prevention. This is unsurprising, because Indonesian educational policy on sexual health focuses on abstinence, rather than health promotion or prevention. One young newcomer described how sexual health was still perceived as taboo, even in high schools:

- Cantik:
- Q: But you said that you graduated from high school, how could you not know about condoms?
- A: No, because I studied at *Pesantren* (a general term for Muslim schools)
- Q: Ohhh
- A: Yes at *Pesantren*, you didn’t discuss condoms at all. The subjects all related to morality and religion.
- …
- Q: Okay, but usually they teach biology and you learned about reproductive matters there, didn’t you?
- A: Yes there was a biology subject but they never mentioned condom protection.

Our data also indicate that despite most newcomer FSWs being married before entering sex work, they did not have a positive orientation towards condom use. Quotes from two newcomers exemplify this:

- Mira:
- Q: Okay, at that time (when you came to a brothel for the first time), did you know about condoms?
- A: No,
- Q: But you were married before…
- A: No, no, never. I didn’t use condoms with my husband.

Kanti:

- A: I never slept with anyone before, only my husband.
- Q: But you could still use condoms with him?
- A: No, never. I never used condoms with my husband. Why should I? He is my husband.
- Q: Don’t you know that the condom is also for contraception?
- A: No, that’s why I didn’t know about condoms before.

There are two important issues from these quotations. First, it seems that condom use was not commonly practised in FSWs’ previous marriages, and second, it is also interesting to note that condoms were not perceived as a contraceptive method. The unpopularity of condoms for contraception among villagers in Indonesia [39] is an old fact that seems to persist. Two further quotations underpin the saliency of this problem. Arik explained that her background as a villager was the reason for her lack of experience and knowledge of condom use. In addition, Kanti (a newcomer) blamed the euphemistic marketing of condoms on television as the reason for her lack of knowledge of condom use.

Arik:

- Q: Before that, had you used condoms?
- A: No, my first condom use was in this location
- Q: Okay, before that had you seen them? In Java maybe?
- A: Much less there. I am from a village, we’d never seen them there.

Kanti:

- A: I never knew about condoms before.
- Q: Had you seen them before?
- A: No never, where? There was advertising on TV, but there is no image of condoms at all there.
- Q: Yes, you are right. I wonder why they never show images of condoms in TV advertising, maybe the government doesn’t allow it.
- A: Yes, I’d never seen them before, that is why I didn’t know.

The lack of STI/HIV knowledge and skills and the lack of condom experience led to newcomers’ perceived lack of self-efficacy towards the use of condoms early in their sex work careers. Indeed, many who experienced using condoms for the first time described being afraid, uncomfortable and embarrassed. The following quote illustrates this:

- Subi:
- A: I was afraid.
- Q: Afraid?
- A: And also embarrassed
- Q: Why?
- A: Some of sex workers there told me to use condoms when having sex with clients. I was so confused, I just replied “yes” although I didn’t know what a condom was.
- Q: Didn’t you know about condoms before?
- A: I didn’t
- Q: Never had used one before?
- A: No, never
- …
- A: My first experience with condoms was with a client. We didn’t start immediately, I opened the packet and I blew into the condom. He asked me my reasons for that act. I thought that is the way to put it on. He explained that was wrong and taught me how to put condoms on correctly. He said that it will prevent us from catching diseases and trouble because of unwanted pregnancy. Yes, he told me how to do it.

While the client in the quote above was ‘good’ in that he educated the newcomer FSW, many newcomers faced clients who tried to negotiate sex without a condom. We can see this in the following extract:

- Arik:
- A: Three days after I arrived I started to work
- Q: Okay, on the first day, did you get some clients?
- A: Oh yes.
- Q: Did they use condoms?
- A: Some did, some not. I didn’t understand, they said that was okay not to use condoms. I told my boss after that, and she yelled at me “Stupid!” On the second day I had an army man as my new client, he forced me to go to the room, he carried me there.
- Q: Carried you?
- A: Yes. “Let me in” he forced. I said no but I was afraid, I couldn’t refuse, I was desperately hopeless at that time.

### Competitive working environments; newcomers as a threat

The situation for newcomers is even riskier than simply moving into the sex work field with insufficient knowledge and skills related to HIV prevention. Our findings indicate these women face oppressive working environments that adversely influence their vulnerability to HIV. From the seniors’ perspectives, newly arrived sex workers (newcomers and mobile women) mean more people competing with them. Many FSWs complained about the shortage of clients and the difficulties of attracting new clients:

- Ayu:
- A: Yeah, you know, recently it’s very hard for us to attract customers, competition is so intense, too many girls.
- …
- There are not many clients now, 50 girls and only 25 men.

Kamel:

- A: Yeah maybe, one thing, if there is a new girl, one that just came to work here, at least she will earn more than us. Maybe yes, in our heart, we feel bit jealous, it’s hard to describe, you know....

Meanwhile, from the newcomers’ perspective, competition is even harder because they have yet to develop regular clients and must also try to understand the ‘rules of the game’. All newcomers revealed that they started work immediately after arrival and their situation as new young girls was maximized to gain economic benefit, partly for them and partly for the pimps. In general, new girls had more clients during their first days. Indeed, one newcomer, Mira, explained that on one night she was the only girl having clients:

- Leni:
- Q: When did you arrive there?
- A: On Saturday.
- Q: Saturday?
- A: and… I started to work on the next day, Sunday.
- Q: When you arrived, was it at night or during the day?
- A: In the afternoon.
- Q: and… you started to work the next day, which is Sunday?
- A: Yes

Mira:

- A: The other sex workers, none of them had any clients, only me. After that they argued with me, they were jealous of me. I went back to my apartment immediately.

Because of their status as new girls, many of them reported receiving an unfriendly reception during their first weeks working as FSWs. One newcomer described feeling like an *asing (*in English, outsider), someone that was both new and unwanted by the senior group:

- Kanti:
- A: Yes, when I arrived, I felt that I was *asing* (an outsider). I am an easy going person, so I felt very weird with that condition. I didn’t know, maybe these women who have worked longer than me disliked my presence, they looked a bit cynical.
- Q: How did you get that feeling?
- A: When they talked, what do you call it? *Nyelengetin* (satirical)
- …
- A: I just laughed. I said “oh yes”, but the analogy is the difference between what your face is indicating and what your heart is feeling. You know! To everyone, I have tried to hide that, I always showed them this face (she smiled).

Some newcomer FSWs experienced serious intimidation by senior FSWs, including warnings to avoid soliciting senior FSWs’ clients or turf, negative talk, magic or seeking shaman help to gain and maintain clients. For example:

- Lusi:
- A: You know, I am new here, compared with other workers. I didn’t talk much. I never did this before. I’m very new and I don’t want to discuss it with others, so I choose to be quiet. But that means clients could recognize me as a newcomer and many of them, including other sex workers’ ex-guests, will try to approach me and that’s the problem. I was accused of hijacking others’ customers. It’s competitive work.

Leni:

- A: Every time a man approached me, they warned me to leave; they said that those men were their boyfriends.

Asih:

- A: When I got back from my village, I found salt in front of my room door.
- Q: In the [brothel] location?
- A: Yes, in front of my working room
- …
- Q: How much?
- A: Quite a lot. It was also in my room.
- …
- A: I think it was one of the women there. Once a client had a transaction with me, then he was with her but probably he just didn’t want her again. He became my regular and she said negative things about me. Another girl told me that.

The harsh competition between FSWs as expressed above has two potential results. First, it forces women to move to another brothel and hence contributes to the instability of brothel-based FSWs. For some respondents moving locations is a strategy to escape from the harsh competition at a brothel, but at the same time it is also a strategy to gain economic benefit as the new girl in town. Ayu spoke about the psychological reasons behind her decision to move to her current working place, while Subi revealed her perception about the economic advantage of moving to another location to attract clients.

Ayu:

- Every day, they mocked me. I was marked as a cheap whore; they said I can’t get any clients. I felt embarrassed but I couldn’t fight back. I couldn’t stay any longer and I didn’t want to fight with them anymore. I didn’t want to make my boss uncomfortable, so I decided to move to my current place.

Subi:

- A: Here she can’t get any clients, but at another place, she’d become a new girl and many clients will look after her there, for sure.
- Q: Hmmm, okay…
- A: Yes, poor girl

The second, more serious consequence was a situation where bargaining for unsafe sex with clients became a viable economic strategy. As one newcomer described, in a situation where she had to choose, she preferred immediate economic benefit over the risk to her health:

Mira:

- Q: Why did you accept him without using condoms?
- A: He said that he didn’t have any diseases.
- Q: And did you trust him?
- A: The truth? No, not really, but with not many clients around I just let him in.

The combination of newcomers’ characteristics and the highly competitive working environment indicates that the women need substantial support to minimize risks and work safely. One approach that might be appropriate to help women work together and set agreed values for safer sex is the concept of empowerment, used in the Sonagachi project in India [40, 41]. However, this type of approach needs a strong sense of social networks and support in the form of social capital. The next section explores this in more detail, with a view to identifying possible interventions based on social capital.

### Lack of perceived social capital in sex worker communities

With the harsh competition experienced by newcomers from their first days as sex workers, it is not surprising to find that newcomers claimed there was a lack of social networks, social support or solidarity between FSWs. There was no sense of FSWs being a community where they could gather and discuss problems and possible solutions or share information about HIV prevention. Additionally, by entering sex work which is viewed as a source of immorality and “dirty” diseases by society in general [18], newcomers felt stigmatized by their work and hesitated about associating with groups of sex workers. They internalized the stigma. For example, Lusi, a high school graduate, claimed she was not part of the sex worker community. She used term *tidak pantas* (in English, inappropriate) to refer to sex work, and hence distanced herself from the women who worked in the same *wisma*. For the same reason, another newcomer, Karmila, described her work as committing a sin, something that she should keep to herself.

Lusi:

- A: I felt *tidak pantas* (inappropriate). Even though I do what they do, I will never associate myself with them. I will not share anything with them; I just feel it isn’t appropriate. From day one I felt that someone here disliked my presence.
- Q: How?
- A: That is normal, in that kind of place. They were also unfriendly towards me…it all makes me feel I don’t want to work there anymore.

Karmila:

- A: I am afraid of sin.
- Q: Sin?
- A: Oh, yes. No one knows that I work this way, the purpose is not this way. That’s why I don’t want to be seen this way.

In 2008, a local NGO started activities to develop networks among FSWs by developing peer group support. However, as indicated by our findings, the programmes struggled to provide meaningful activities to attract more FSWs into the network. Under agreements made between pimps, local area authorities, the local health office and the local NGO, health education sessions were delivered on a regular basis at each brothel. These sessions became the major social activity that brothel-based FSWs could participate in. A senior FSW, who was appointed a peer educator by the local NGO, reported a lack of interest from FSWs:

Ayu:

- A: The difficulty is they don’t want to.... I tried and failed. Based on my experiences, every time I asked them, they said: “take care of yourself, don’t bother me, do your own business.” Many of them said that.

The reluctance to attend sessions was also reported by FSWs. For example, while some newcomer FSWs saw opportunities to make connections with their peers, senior FSWs actively discouraged such networking:

- Kanti:
- A: I met other girls during my blood test a couple of weeks ago; all girls from different houses were coming to be tested. That was my first time, so when I met them, I put a big smile on my face and tried to be polite by saying ‘excuse me’. However, what a surprise! No one replied, they looked cynical, they looked like they didn’t like me as I was new. I saw them whispering, maybe talking about me. I was shocked. When I finally got back, I asked my peers from my working place and they replied as if I was stupid. They were questioning my effort to get to know other girls from different houses; they claimed that my intention was useless.

Another reason that may significantly contribute to the reluctance of FSWs to participate in activities delivered by NGOs or other health providers relates to the timing of activities, which has never been acknowledged nor addressed by current health promotion programmes in Bali. Findings indicate that FSWs have limited free time during the daytime when most health education activities are conducted. Most FSWs claimed that they needed to work until very late at night, sometimes until 2–3 am. In addition, some of them worked both during mornings and at night, leaving only the afternoon as a time to get rest. Attending FSW health education groups was very low on their priorities to consider.

Ratna:

- Q: Not really?
- A: We are tired.
- Q: Tired?
- A: We work until late at night. If we had a group and spent our time there during the day… that would be hectic. I use my spare time to have a rest.

The majority of participants considered that few significant benefits accrued from groups or social activities involving FSWs, but we found some women who were involved in money saving groups (they use term *arisan*). Although our findings suggest that this type of group did not provide enough opportunities for FSWs to gather and conduct social activities, many sex workers considered them beneficial, particularly in helping them to save money. Ari was one newcomer who spoke of the benefits of joining an *arisan*.

Ari:

- Q: Why did you join an arisan?
- A: In this kind of working environment, you really need to save money. You don’t want to waste it.
- Q: Why?
- A: I don’t know, sometimes we spent our money hastily. You know, money that you get from this job, it is easy to spend it.

At this point, we have shown the nature of the social networks of FSWs in Bali, characterized by intense competition and lack of social or group activities. Because of this, most newcomers also confirmed the lack of perceived trust and support with and from other FSWs. An additional concern is that some newcomers also distrusted any ‘support’ offered by their seniors, often viewing them as having ulterior motives. This sense of conspiracy was often founded on bad experiences. For example, Mira, a young newcomer, claimed that some seniors ruined her reputation by using their knowledge of her STI history. She admitted that on many occasions she had been treated well by her seniors; however, she remained suspicious regarding the motives behind the offered support.

Mira:

- A: When I knew that I was infected, they mocked my problem, I was so ashamed. They asked clients not to sleep with me; they told them that I was infected.
- …
- Yes, there are some that treated me nicely, but I know that’s only on the outside.
- Q: Hmm. What do you mean by that?
- A: Yes, they said that they support me, but then when I wasn’t around they mocked my problem in front of others.

To further understand the lack of perceived trust and support identified by newcomers, the study also explored senior FSWs’ perceptions regarding their willingness to help and support newcomers to adapt to the working conditions in safety. Our findings indicate that support from senior FSWs is conditional. The first condition is whether they trust the motives and history of the newcomer. As previously discussed, moving to a new brothel is perceived as a strategy to gain economic benefit as a new girl, and some senior FSWs expressed their hesitance to provide help to a new girl because they did not know her history and whether she was a real newcomer or a mobile FSW.

Sandra:

- A: The problem is a new girl is not always truly a new girl directly from Java. She could also be someone with experience who has come from another brothel. Many of them are mobile; hence, I don’t want to explain how to work as a sex worker or how to use condoms because she might know better than me.

The second condition related to perceived economic threat. One senior FSW claimed that newcomers who were popular with clients received less support and could be subjected to unpleasant treatment by other FSWs designed to create an unfriendly working environment:

Ratna:

- A: Sometimes we help them; however it depends on whether she is beautiful or popular. If so, most of the time other sex workers will abandon her and treat her as an enemy.
- …
- A: So she will never feel comfortable working there.
- …
- A: Finally, she’ll move again.

The support withheld by senior peers extended to important health and safety issues such as how to deal safely with clients, handling physical violence, or negotiating condom use. Many FSWs shared unpleasant and unsafe experiences about first-time sexual transactions, once again highlighting this group’s vulnerability to STIs and HIV:

- Cantik:
- A: Yeah, if we have never accepted a particular client before, you hardly know if he is good or bad…you’ll know in time
- Q: Oh so…
- A: I experienced being treated badly inside the working room, not all guests are kind.
- Q: Didn’t your seniors inform you before, give you information on how clients behave?
- A: No one warned me about that.

Kamel:

- A: When I was new, no one told me how to work safely, no one, we don’t care for each other, we are apathetic.
- Q: Really, no one tells others?
- A: No one, some yes, but only about how much they usually get paid
- Q: Only that?
- A: Ho-oh. Yes that’s it, other matters, they won’t tell
- Q: Something related to health?
- A: No, no one.

Yes that’s crazy. The thing that they tell you is the price range … don’t be long, make it quick and if the client wants longer time, charge them double, that’s it.

During my first weeks there, no one told me to use condoms or even offered me a condom. I didn’t know what condoms looked like, so when I had clients I just did it, simple and crazy, really crazy.

## Discussion

The findings of this study have demonstrated that the sex work industry in Bali as a new social field for newcomers and mobile women is a hazardous environment fraught with risks of HIV. These groups become more vulnerable to HIV infection because of the interaction of a number of social factors. First, epidemiologically, newcomer FSWs have little knowledge and few skills related to STI and HIV prevention, but are exposed to unsafe sexual activities with clients. Consequently, Bali is ranked as one area in Indonesia with high prevalence of HIV among FSWs [7]. Second, newcomer FSWs are socially vulnerable, because they are not involved effectively in social networks that are able to provide access to sufficient social support in order to work safely as FSWs. Third, the women turned to sex work to maximize economic benefits, and this situates newcomer FSWs in a precarious economic and health-related situation. Given their lack of regular clients and knowledge of the ‘rules of the game’, newcomers have to choose between committing to condom use (and thus losing potential income but reducing HIV risk) or allowing clients to not wear condoms (which increases financial gain but also increases HIV risk). Efforts to maximize economic benefit sometimes involved a strategy to damage other FSWs’ reputations and thus reduce the willingness of clients to pay for sex with them, which further undermines the potential for developing social capital.

In this paper, we have highlighted that structural inequalities play out at the micro-structural level; in this case, social networks of FSWs. Newcomers consciously have to exercise their agency by negotiating new identities in the context of structural inequalities. Condom use is negotiated with clients and thus consistent condom use is not seen as the major priority. FSWs are in a competitive environment whereby the main objective is to survive in the short term with the hope of earning enough income to enable them to leave sex work and return to their villages, often in Java. In the absence of social support and reciprocity from other FSWs, risk as embodied through not using condoms becomes normalized and therefore is no longer a ‘risk’ to be avoided or remedied [42]. Interventions that aim to change these micro-level structural factors must first remove barriers to social network development [43]. The question becomes how to empower FSWs in Bali to engage in social network building activities with a view to developing a sense of social capital, reciprocity, trust and solidarity. Even with this, we need to keep in mind the social stigma of FSW which often makes FSWs reluctant to associate with sex worker networks or organizations [44].

Community empowerment has been suggested as a key mechanism and philosophy to solve some of the seemingly intractable problems outlined above. Community empowerment would aim to provide FSWs with agency and the means to control their decisions, but it depends on the strength of the existing social network structure. A strong social network may provide sufficient support to reinforce positive values of safe sex in FSW communities. Referring to the Sonagachi model, the best practice example of community mobilization among FSWs in India, community-led interventions can be remarkably successful if projects address the needs of the community, involve grassroots participants in leadership and enhance social capital [40]. Interventions fostering community change should view social groups or networks of FSWs as the agents of change. These groups usually operate within defined geographic areas, and the process of change is delivered by a combination of social influence or diffusion within these groups (requiring change champions) and within the particular social and physical environments in which the risk behaviour occurs, such as a brothel. In this way, social support and influence will be available for FSWs already in the field and also for newcomers to FSW.

However, the question remains. Where to start? Activities involving FSWs in HIV prevention in Bali have been conducted for a number of years, yet as shown by the findings of this study, they have failed to develop sufficient dimensions of social capital among FSWs. Community empowerment relies on people feeling that they are part of a community; it is clear from our findings that most FSWs did not perceive good community cohesion, while some of them even refused to be associated with sex workers as a group or profession. Looking again to the Sonagachi programmes for inspiration, health is communicated in relation to the sex workers’ context of living. For example, one campaign for sex worker health emphasizes a mother who needs to stay healthy to maintain the family. In Bali, this might also be the case. The majority of FSWs are workers from other islands in Indonesia, with expected roles as devoted mothers to their children or loyal daughters to their parents. Our findings also showed that as migrant workers they bear familial expectations of financial success to support family members in their villages. New community led interventions to build social capital might therefore start with social activities that maximize the economic capital of FSWs. Our findings about women’s interest in money saving groups could be the starting point.

A number of studies have also highlighted socio-economic pressure as an important factor that constrains the scope and pace of community empowerment programmes [45–48]. Hence, recognising that limited access to resources in affected communities increases their social vulnerability to HIV, projects in Kenya and India included micro-finance programmes or banking co-operatives to allow women to save money and get loans at reasonable rates [41, 49–51]. Such economic development programmes help the most affected communities to maintain financial security and provide an alternative livelihood and hope for the future [49, 52]. We believe that there is the potential for similar groups to be included in community empowerment programmes for FSWs in Bali. This is a different course to the dominant biomedical public health strategy in Bali and Indonesia, where the focus is on meeting ‘normative needs’ [53, 54], or what professionals ‘think’ FSWs ’need’. Such normative needs may focus on providing health education related to the accessibility and use of condoms, instead of the actual priorities and felt needs of FSWs. However, based on our findings we urge policy makers and practitioners to develop programmes that address the lives of FSWs more holistically, and to be responsive to their priorities.

## Conclusion

In examining social capital in the context of sex work in Bali we have demonstrated how newcomers’ social characteristics and their struggle to exercise their agency in a new social field shapes their vulnerability to HIV, and how this represents a new opportunity for more effective intervention efforts. Our data show that moving into sex work, often after migrating from villages and education settings where sexual health is not talked about, creates the first level of HIV vulnerability. This is compounded by additional vulnerabilities based on the competitive environment of sex work and economic rationality. FSWs become competitors in a sex marketplace, trying to increase their market share and profitability while also trying to reduce the profitability of ‘competitors’. Developing social capital, trust, reciprocity and solidarity in this cut-throat environment becomes difficult; indeed, it potentially undermines women’s access to economic capital. In this marketplace, the price for sex without a condom is much higher than for safer sex. This places FSWs in a double-bind: saying ‘no’ to clients who do not want to use condoms has short-term (losing this transaction) and long-term (losing current and future clients to another FSW who will say ‘yes’) economic disadvantages. However, saying ‘yes’ to these clients may be financially beneficial in the short term, but increases the risk of HIV. These are the real-world concerns of newcomer FSWs in Bali. Any new approach to HIV prevention must take account of women’s voiced concerns and needs as well as specific opportunities and constraints in the socio-political lives of FSWs.

## Authors’ information

PPJ is a Balinese public health physician. He has been working in HIV prevention programmes in Bali since 2009 and is currently undertaking a doctorate in public health at Flinders University, Adelaide, Australia. JM is a senior lecture in qualitative health research at the Centre for Values, Ethics and the Law in Medicine, University of Sydney. She is the director of the Sydney Qualitative Health Research postgraduate coursework programme and undertakes health services research with a focus on stigmatized conditions and marginalized populations. PRW is Professor of Public Health and Associate Dean (Research) in the Faculty of Medicine, Nursing and Health Sciences at Flinders University, Australia. He is a sociologist with particular interests in applying social theory and innovative methods to public health issues.
